# Citrate Synthase Expression Affects Tumor Phenotype and Drug Resistance in Human Ovarian Carcinoma

**DOI:** 10.1371/journal.pone.0115708

**Published:** 2014-12-29

**Authors:** Lilan Chen, Ting Liu, Jinhua Zhou, Yunfei Wang, Xinran Wang, Wen Di, Shu Zhang

**Affiliations:** 1 Department of Obstetrics and Gynecology, Ren Ji Hospital, School of Medicine, Shanghai Jiao Tong University, Shanghai, 200127, P. R. China; 2 Shanghai Key Laboratory of Gynecologic Oncology, Shanghai, 200127, P. R. China; Wayne State University School of Medicine, United States of America

## Abstract

Citrate synthase (CS), one of the key enzymes in the tricarboxylic acid (TCA) cycle, catalyzes the reaction between oxaloacetic acid and acetyl coenzyme A to generate citrate. Increased CS has been observed in pancreatic cancer. In this study, we found higher CS expression in malignant ovarian tumors and ovarian cancer cell lines compared to benign ovarian tumors and normal human ovarian surface epithelium, respectively. *CS* knockdown by RNAi could result in the reduction of cell proliferation, and inhibition of invasion and migration of ovarian cancer cells in vitro. The drug resistance was also inhibited possibly through an excision repair cross complementing 1 (ERCC1)-dependent mechanism. Finally, upon *CS* knockdown we observed significant increase expression of multiple genes, including *ISG15*, *IRF7*, *CASP7*, and *DDX58* in SKOV3 and A2780 cells by microarray analysis and real-time PCR. Taken together, these results suggested that CS might represent a potential therapeutic target for ovarian carcinoma.

## Introduction

Ovarian cancer is one of the leading causes of mortality in malignant gynecological tumors. There were approximately 22,240 new cases and 14,030 deaths associated with ovarian diseases in the United States in 2013 [1]. One reason for this high mortality rate is that ovarian cancer is often diagnosed at late-stage. Surgical resection and subsequent chemotherapy are still the major therapeutic strategies, with limitation for controlling cancer growth and metastases [2]. Furthermore, drug resistance and cancer recurrence are major clinical challenges.

The tricarboxylic acid (TCA) cycle regulates energy generation in mitochondrial respiration and plays a central role in carbohydrate metabolism. Citrate synthase (CS) catalyzes the first reaction of the TCA cycle and is generally assumed to be the rate-limiting enzyme of the cycle [3]. Increasing evidence suggests that CS activity is closely associated with various kinds of cancers. The activity of citrate synthase was measured using tissue extract prepared from specimens (pancreatic cancer and control specimens taken from the adjacent pancreatic normal tissue) obtained from 24 patients with ductal carcinoma who underwent pancreatoduodenectomy or total pancreatomy, enhanced CS activity was observed in pancreatic cancer [4]. It is likely that enhanced citrate synthase activity contributes to the conversion of glucose to lipids in pancreatic cancer providing substrate for membrane lipids synthesis. In an in-vitro model, Ramos cells (Burkitt lymphoma cell line) were exposed to varying concentrations of doxorubicin and vincristine for 1 hr; and allowing for recovery in culture over a 7-day period, recovering or residual cells from chemotoxicity exhibited an increase in citrate synthase [5]. All these suggested CS play an essential role in tumors.

The activity of CS in ovarian cancer has been studied previously. Anderson et al. used the mouse ovarian surface epithelial (MOSE) cancer progression model to study metabolic changes in distinct disease stages, they found citrate synthase activity was increased during the ovarian cancer progression [6], however, the expression of CS in human ovarian tissues was not investigated. In current study, based on observation that high expression of CS in malignant ovarian tumors and ovarian cancer cell lines, we hypothesized that CS might contribute to the ovarian cancer phenotype as well as drug resistance and thus represent a therapeutic target. We evaluated the consequences of transient *CS* inactivation by silencing the *CS* gene in SKOV3 and A2780 ovarian cancer cell lines. Here, we provided the first demonstration that inactivation of CS result in defective cell proliferation, cellular invasion, migration, and increased chemosensitivity in ovarian cancer cells. Additionally, microarray analysis of *CS*-inactivated cells identified several candidate genes that might promote tumor chemosensitivity, apoptosis, and autophagy.

Taken together, these findings suggested that CS play an important role in regulation of ovarian cancer cell proliferation, invasion, migration, and chemoresistance and indicated that inhibition of CS could be a potential therapeutic strategy for treatment of ovarian cancer.

## Materials and Methods

### Tumor specimens and Ethics Statement

A total of 32 tissue specimens were collected from surgical patients enrolled in the study, including 21 primary epithelial ovarian cancer (EOC) tissues and 11 benign ovarian tumor tissues. All specimens were obtained during surgery (Department of Obstetrics and Gynecology, Ren Ji Hospital, School of Medicine, Shanghai Jiao Tong University, China), frozen immediately in liquid nitrogen, and stored at −80°C until analysis. All neoplasms were primary ovarian tumors, and no patient received chemotherapy prior to surgery. The study was approved by the Institutional Review Board of Ren Ji Hospital, School of Medicine, Shanghai Jiao Tong University, and written informed consent was obtained from all patients. All clinical investigation was conducted according to the principles expressed in the Declaration of Helsinki.

### Cell culture

Human ovarian adenocarcinoma cell line SKOV3 and A2780 cells were obtained from the Chinese Academy of Sciences and cultured in RPMI 1640 (Hyclone) medium supplemented with 10% fetal bovine serum (Gibco). Cells were incubated at 37°C in a humidified atmosphere of 5% CO_2_. The medium was replaced every 24 h.

Normal human ovarian surface epithelium (HOSE) was obtained from ovarian biopsies at laparoscopic procedures for nonmalignant gynecological conditions. For biopsy specimens, hold the entire specimen surface down over a 60 mm culture dish, then wash the specimens with sterile PBS for four times, and scrape the ovarian surface gently several times with a scraper. The mixture of scraping was transferred to sterile centrifuge tube and centrifuged (800 rpm) for 3 min. The sediment was mixed with Collagenase (Serva) for 5 min at 37°C with 5% CO_2_. Then the mixture was centrifuged (800 rpm, 3 min) and the supernatant was transfered to 25 cm^2^ culture flask. Primary cells were cultured in DMEM/F12 (Hyclone) medium supplemented with 15% fetal bovine serum and 100 units/ml penicillin and 100 g/ml streptomycin (Sigma) and cells were incubated at 37°C in 5% CO_2_. Normal HOSE cells were sorted by FACS Aria cell sorting system with EpCAM conjugated with PE (eBioscience) as an epithelium marker. After sorting, the HOSE cells were cultured in 60 mm dish about 12 days, the medium was replaced each 3 days. mRNA and protein were extracted from the HOSE cells.

### Transient transfection of SKOV3 and A2780 cells with CS siRNA oligonucleotides

Transient small interfering RNA (siRNA) oligonucleotides were designed and synthesized from Shanghai Integrated Biotech Solutions Co., Ltd. The target sequences were as follows – siCS1078: 5′-*GCG AGA GUU UGC UCU GGA AAC A*-3′; siCS1122: 5′-*AGU UGG UUG CUC AGC UGU ACA*-3′. As a control for silencing, we constructed a negative control (NC) siRNA (5′-*UUC UCC GAA CGU GUC ACG UTT*-3′) that did not affect CS expression. Transient transfections were carried out on 40% confluent cell cultures in 6-well plates using Lipofectamine 2000 Transfection Reagent (Invitrogen, Carlsbad, CA) according to the manufacturer's instructions. RNA was extracted after 24 h and total protein was collected after 48 h.

### RNA extraction and quantitative real-time reverse transcriptase PCR

Total RNA was extracted from SKOV3 and A2780 cells, normal human ovarian surface epithelial cells, and human ovarian tumor samples using TRIzol Reagent (Invitrogen). Total RNA was reverse transcribed with a PrimeScript RT reagent Kit (TaKaRa, Shanghai, China) according to the manufacturer's instructions. The resultant complementary DNAs (cDNAs) were used for quantitative real-time reverse transcriptase PCR using a SYBR Green PCR Master Mix Reagent Kit (TaKaRa) according to the manufacturer's protocol. Real-time PCR and data collection were performed on an ABI 7500 real-time system (Applied Biosystems, USA). The primers for *CS* were 5′-*GAT TGT GCC CAA TGT CCT CT*-3′ (forward) and 5′-*TTC ATC TCC GTC ATG CCA TA*-3′ (reverse). The primers for *ISG15* were 5′-*CGC AGA TCA CCC AGA AGA TCG*-3′ (forward) and 5′-*TTC GTC GCA TTT GTC CAC CA*-3′ (reverse). The primers for *IRF7* were 5′-*GCT GGA CGT GAC CAT CAT GTA*-3′ (forward) and 5′-*GGG CCG TAT AGG AAC GTG C*-3′ (reverse). The primers for *DDX58* were 5′-*CTG GAC CCT ACC TAC ATC CTG*-3′ (forward) and 5′-*GGC ATC CAA AAA GCC ACG G*-3′ (reverse). The primers for *ATG12* were 5′- *CTG CTG GCG ACA CCA AGA A*A-3′ (forward) and 5′-*CGT GTT CGC TCT ACT GCC C*-3′ (reverse). The primers for *β-actin*, the endogenous control gene, were 5′-*TGA CGT GGA CAT CCG CAA AG*-3′ (forward) and 5′-*CTG GAA GGT GGA CAG CGA GG*-3′ (reverse). SDS v.1.4 (Applied Biosystems) software was used to perform comparative delta cycle threshold (Ct) analysis. To minimize variation in experiments, each real-time PCR experiment was performed in triplicate.

### Citrate synthase activity assay

Ovarian cancer cells (48 h post-transfection) were used to examine citrate synthase activity according to the protocol provided by citrate synthase kit (Beijing Solarbio Science & Technology Co., Ltd, China). Protein level was quantified by BCA kit (Cwbiotech, China).

### ATP assay

Intracellular ATP concentrations of ovarian cancer cells (48 h post-transfection) were determined by phosphomolybdic acid colorimetric method (Nanjing Jiancheng Bioengineering Institute, China) according to the manufacturer's protocols. Protein level was quantified by BCA kit (Cwbiotech, China).

### Cell proliferation assay

48 h after transfection, cell proliferation was assessed by the Cell Counting kit-8 (CCK-8) (Dojindo, Japan). Briefly, 2×10^3^ cells were seeded into 96-well plates. For the next 3 days, 10 µl CCK-8 was added to each well for 1.5 h at 37°C on each day. The absorbance (450 nm) was measured using a microplate reader (Multiscan MK3; Thermo Scientific, USA) at the indicated time points.

### Cell invasion and migration assay

Ovarian cancer cells (6×10^4^) (48 h post-transfection) were placed into the upper compartment of the chambers (8 µm pore size, Millipore) coated with 50 µl BD Matrigel (diluted 1∶7 in serum-free medium), chambers were then placed into 24-well plates. Medium containing 10% fetal bovine serum was added to the lower chamber. After incubation at 37°C for 24 h, cells on the upper side of the membrane were removed using sterile cotton swabs. Cells adhering to the lower surface were fixed in 4% paraformaldehyde and stained with 0.1% crystal violet. Cells were counted using light microscope (Nikon TE300, Tokyo, Japan) at 200× magnification. Five random fields were selected for examination on each membrane, and results were expressed in terms of the invasion cells per field. Each experiment was conducted in triplicate. Cell migration was performed using a similar approach without Matrigel coating, and the treated cells were incubated for 12 h.

### Western blot analysis

Cells were lysed on ice for 30 min using RIPA buffer and PMSF (Beyotime, Shanghai, China). Proteins were separated by sodium dodecyl sulfate (SDS)-polyacrylamide gel electrophoresis and transferred to a nitrocellulose membrane or a polyvinylidene fluoride membrane (Millipore, Billerica, MA). After blocking with 5% dry milk in Tris-buffered saline with Tween-20 (TBST) for 2 h at room temperature, the membranes were incubated with primary antibodies against human CS (1∶5000, Epitomics, Burlingame, CA), β-tubulin (1∶4000, Epitomics), β-actin (1∶500, Abmart), GAPDH (1∶4000, Abmart), p38 (1∶1000, Cell Signaling Technology, Shanghai, China,), p-p38 (1∶1000, CST), ERK (1∶1000, CST), p-ERK (1∶1000, CST), Vimentin (1∶1000, CST), ERCC1 (1∶400, Abcam, Cambridge, UK)), γ-H2AX (1∶1000, CST), Bcl-2 (1∶4000, Epitomics), caspase 3 (1∶1000, CST), p-FAK (1∶1000, CST), FAK (1∶1000, CST), MMP2 (1∶500, Abcam), AMPKα (1∶1000, CST), p-AMPKα (1∶1000, CST) in dilution buffer overnight at 4°C. After washing three times with TBST, membranes were incubated with an IRDye 800 CW Conjugated Goat (polyclonal) Anti-Rabbit or Anti-Mouse IgG secondary antibody (1∶10000, LI-COR, Lincoln, USA ) for 1 h at room temperature. Protein expression was detected using the LI-COR Odyssey Infrared Imaging System (Biosciences) following the manufacturer's instructions. The relative grey of each protein was analyzed by Image J software.

### Drug sensitivity analysis

Ovarian cancer cells (5×10^3^/well) were cultured in 96-well plates with *CS* siRNA. After 48 h, cells were incubated with different concentrations of cisplatin (DDP; Sigma) for another 24 h. The median growth inhibitory concentration (IC50) was determined by CCK8 assay. SKOV3 and A2780 cells were transfected the same way as above in 6-well plates, after 48 h, total protein was extracted and expression of ERCC1 and γ-H2AX was analyzed by Western blot analysis.

### Colony formation assay

Ovarian cancer cells were treated with indicated concentrations of cisplatin for 12 h or 1 h after *CS* silencing for 48 h. Then cells were plated in 6-well plates at a density of 1×10^3^ cells per well under normal culture conditions for 8 days, the medium was changed every 3 days. Cells were then fixed with 4% paraformaldehyde, stained with 0.1% crystal violet, photographed using a Nikon D80 digital camera, and colonies were counted.

### Microarray analysis

Total RNA was extracted from SKOV3 cells that were transiently transfected with NC or siCS1078 oligonucleotides for 48 h as above, and RNA integrity was evaluated by microfluidic analysis using the Agilent 2100 bioanalyzer with an RNA LabChip Kit (Affymetrix,USA). Only RNA preparations with a 28 S/18 S ratio near 2.0 were used. RNA was reverse-transcribed with the Genechip 3′ IVT Express Kit (Affymetrix). In total, 15 µg of aRNA was hybridized to Affymetrix HG-U133 Plus 2.0 microarray chips. Arrays were washed, stained, and scanned according to standard protocols supplied by the manufacturer. After scanning, files were normalized and compared using the invariant set method according to the Partek software (St.Louis, MO, USA). This comparison identified genes that were upregulated and downregulated in *CS*-silenced cells. The expression of several genes was validated by real-time PCR in SKOV3 and A2780 cancer cells.

### Statistical analysis

All data were presented as the means ± standard error (SEM). An independent Student *t* test was used to compare the continuous variables between groups. *P*<0.05 was considered statistically significant. All statistical analyses were performed using GraphPad Prism 5.0 software (San Diego, CA).

## Results

### CS expression in human malignant ovarian tumors, benign ovarian tumors and ovarian cells

**Figure 1:**
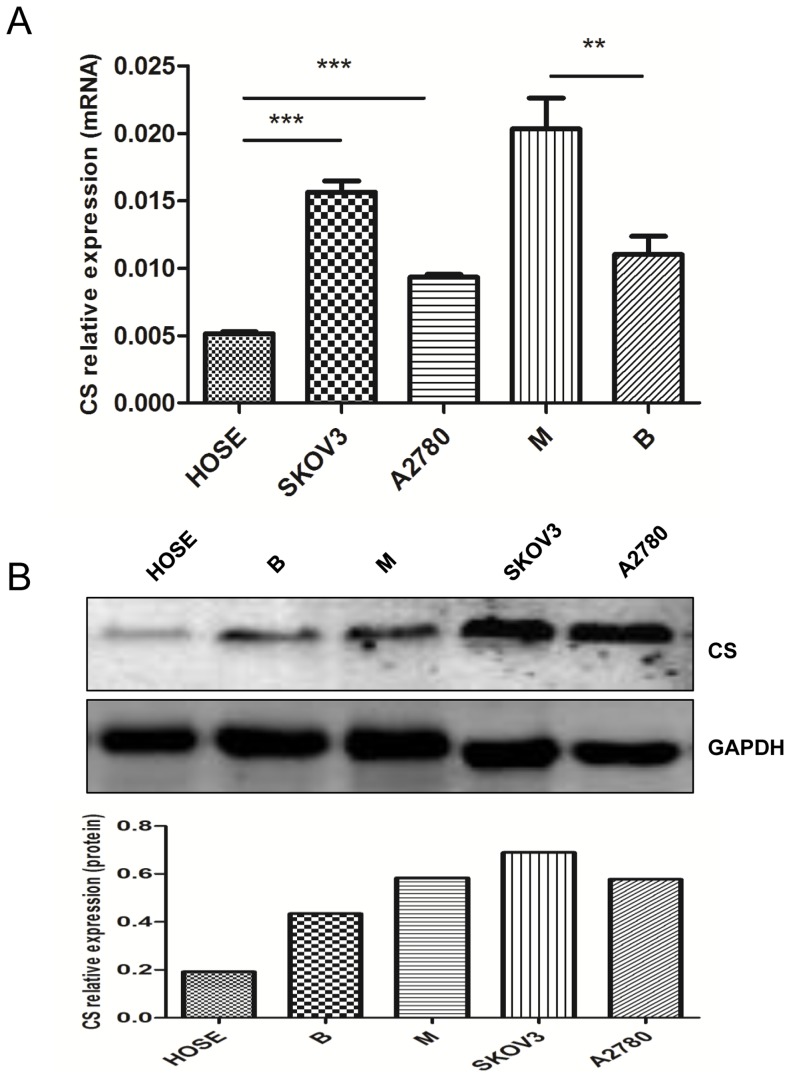
Up regulated expression of citrate synthase (CS) in human ovarian tumors and human ovarian tumor cell lines. (**A**) *CS* mRNA and (**B**) protein expression was assessed in normal human ovarian surface epithelium (HOSE), ovarian cancer cell lines, benign (n = 11) and malignant ovarian tumors (n = 21) using real-time PCR and western blot, respectively (B =  ovarian benign tumor, M =  ovarian malignant tumor). Mean ± SEM. **P<0.01 and ***P<0.001.

In order to find out whether CS was abnormally expressed in human ovarian tissues and cells, we compared mRNA and protein expression of CS in human ovarian carcinoma tissues, ovarian benign tumors and normal HOSE. Results showed mRNA expression of *CS* in ovarian carcinoma tissues was 1.843 times of ovarian benign tumors (*P* = 0.0064) (Fig. 1A), the protein level was also upregulated in human ovarian carcinoma compared with the benign tumors (Fig. 1B). Normal HOSE cells were cultured from normal ovarian epithelium tissues [7], [8], [9] and were sorted by FACS Aria cell sorting system with EpCAM conjugated with PE (eBioscience) as an epithelium marker [10], [11]. The representive images of primary cells were shown in S1A–B Fig. The purity of normal HOSE was about 17% before cell sorting (S1D Fig.). After sorting, the HOSE cells were cultured in 60 mm dish about 12 days, the medium was replaced each 3 days. mRNA and protein were extracted from the HOSE cells (about 3×10^5^ cells) for research. C*S* mRNA and protein expression were also elevated in SKOV3 and A2780 cells compared with normal HOSE cultured from ovarian epithelium tissues (*P*<0.001) (Fig. 1A, B).

### The effect of CS on ATP and AMPK/P38 MAPK in human ovarian cancer cells

**Figure 2:**
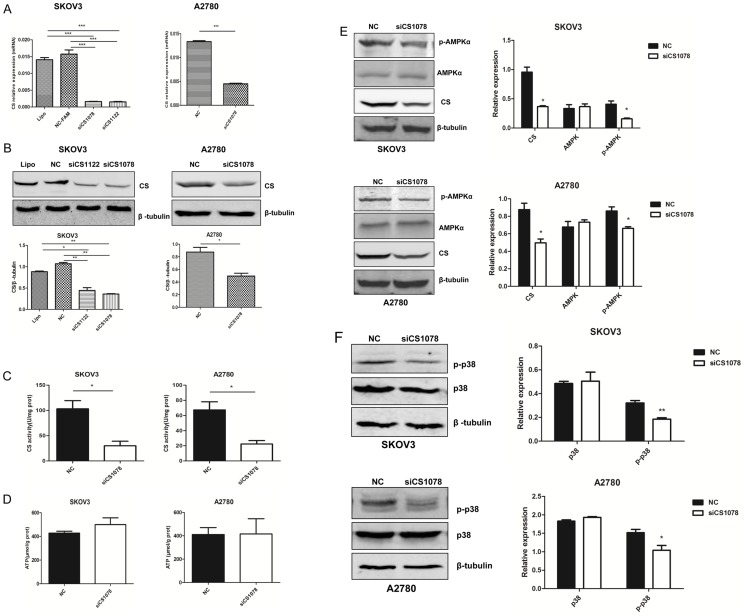
*CS* silencing affects AMPK/P38 MAPK pathway in ovarian cancer cell lines. (**A**) mRNA and **(B)** protein expression level of CS by real-time PCR and western blot in SKOV3 and A2780 cells after *CS* siRNA (100 nM) for 24 h and 48 h after transfection, respectively. (**C**) Decreased CS activity after 48 h transfection in SKOV3 and A2780 cells. (**D**) ATP level was examined 48 h after *CS* silencing. (**E, F**) p-AMPKα and p-p38 were analyzed in *CS*-silenced cancer cells by western blot. Mean ± SEM. *P<0.05, **P<0.01 and ***P<0.001.

In order to study whether the up-regulated expression of CS was correlated with malignant phenotype of ovarian cancer, siRNA was applied on human ovarian adenocarcinoma cell lines. After SKOV3 and A2780 cells were treated with *CS* siRNA for 24 h, *CS* mRNA level was reduced about 88.60% and 66.29%, respectively (*P*<0.001) (Fig. 2A). Under the same conditions, CS protein expression and activity were decreased significantly after 48 h transfection (*P*<0.05) (Fig. 2B, C). Ovarian carcinoma consumes high amounts of energy and TCA cycle produces ATP in the mitochondria. CS is one of the key enzymes in TCA cycle, when CS was silenced in SKOV3 and A2780 cells, ATP level was upregulated in *CS*-silenced cells, though the difference was not significant (*P*>0.05) (Fig. 2D). It might because there was compenastory mechanism to prompte TCA cycle in *CS*-silenced SKOV3 and A2780 cells. AMPK acts as an energy sensor, modulating metabolic stresses such as hypoxia and respiratory impairment, any stimuli that increase AMP or decrease ATP can activate AMPK. The level of p-AMPKα was greatly decreased in the *CS*-inhibited cells compared to NC group (*P*<0.05) (Fig. 2E). In addition, the AMPK/p38 MAPK signaling cascade stimulates glucose uptake during metabolic stress. To confirm the effect of increased AMPK activity in *CS* knockdown cells, phosphorylated p38 MAPK (p-p38 MAPK) level was also examined. The level of p-p38 MAPK was also greatly decreased in the *CS*-silenced cells compared to the negative control cells (*P*<0.05) (Fig. 2F).

### CS silencing results in the reduction of cell proliferation in ovarian cancer cells

**Figure 3:**
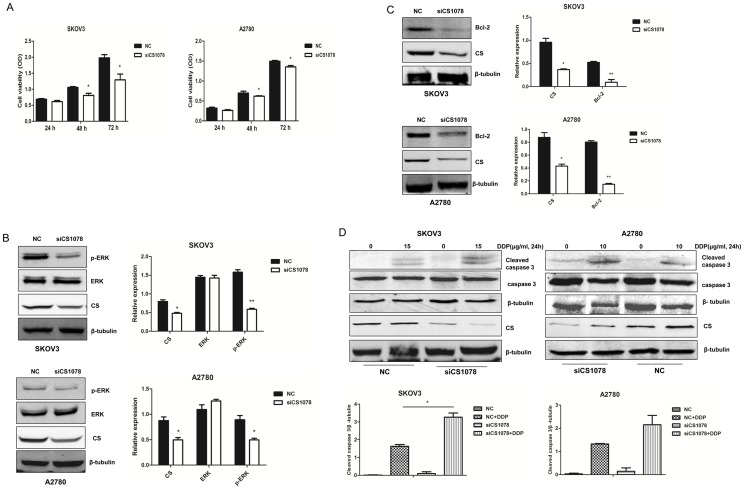
*CS* silencing results in proliferation reduction in SKOV3 and A2780 cells. (**A**) Cell proliferation was measured in *CS*-silenced cancer cells and control groups. (**B**) p-ERK and **(C)** Bcl-2 expression were measured after *CS* siRNA for 48 h in SKOV3 and A2780 cells by western blot. β-tubulin was used as a loading control. (**D**) 48 h after *CS* siRNA, cells were treated with indicated concentrations of DDP (15 µg/mL or 10 µg/mL) for 24 h. Cleaved caspase 3 was measured by western blot. Mean ± SEM. *P<0.05 and **P<0.01.

CCK8 was used to examine the influence of CS on cell proliferation. Ovarian cancer cell proliferation was significantly reduced after *CS* silencing (*P*<0.05) (Fig. 3A). The extracellular signal-regulated kinase (ERK) pathway is a key component in the control of cell growth. This signaling is often upregulated in a diverse range of human cancers. The level of p-ERK was largely decreased in the *CS* knockdown SKOV3 and A2780 cells as compared to the control (*P*<0.05) (Fig. 3B). These results indicated that inhibition of CS expression resulted in downregulation of cell growth signaling in SKOV3 and A2780 cells.

### CS silencing enhances apoptosis in SKOV3 and A2780 cells

Cleaved caspase 3, and Bcl-2 are representative proteins related to cell apoptosis. DDP is a chemotherapy drug used to treat ovarian cancer, it operates by forming a platinum complex inside the cell which binds to DNA and cross-links DNA and causes the cells to undergo apoptosis, or systematic cell death. Cleaved caspase 3 was upregulated in *CS*-silenced cells treated with DDP for 24 h (Fig. 3C, D), especially in SKOV3 cells (*P*<0.05). And Bcl-2 was decreased in *CS*-inhibited cancer cells (*P*<0.01). The results suggested *CS* silencing could promote ovarian cancer cell apoptosis.

### CS silencing inhibits cell invasion and migration in vitro

**Figure 4:**
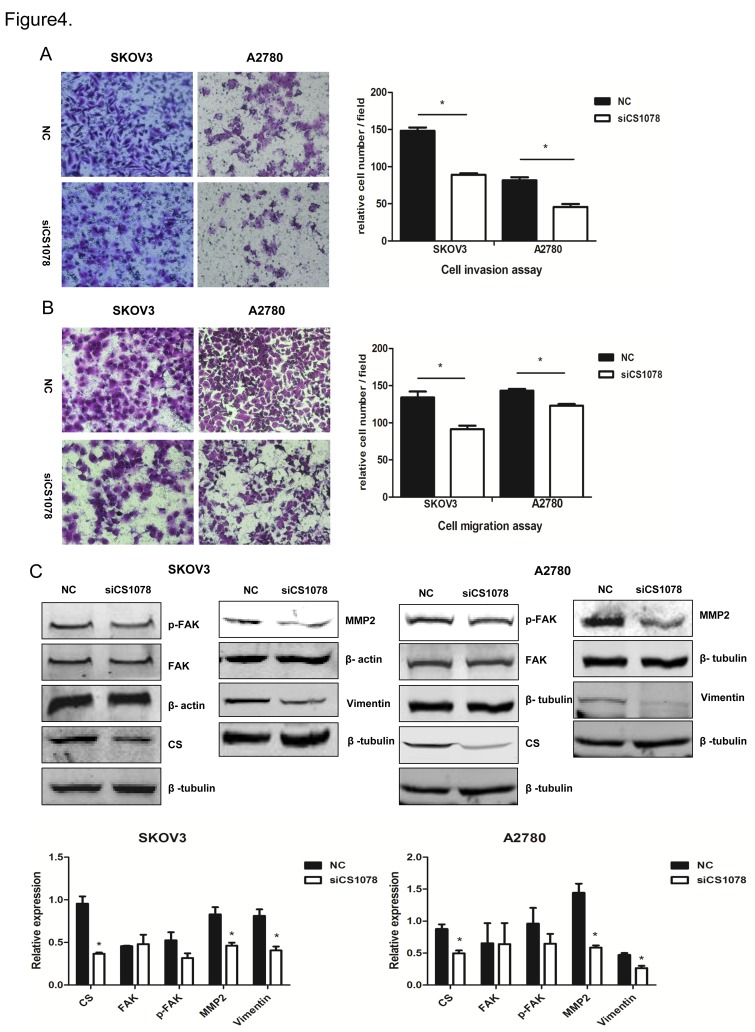
*CS* silencing inhibits SKOV3 and A2780 cell invasion and migration in *vitro*. 48 h after transfection, cell invasiveness was evaluated using the transwell assay and the Boyden Chamber test was used to measure the extent of cell migration. The number of invaded and migrated cells was counted using a bright-field microscope (200×). Representative images and the relative cell numbers were shown in (**A**) and (**B**). (**C**) p-FAK, MMP2 and Vimentin in *CS* knockdown cancer cells were analyzed using western blot. Mean ± SEM. *P<0.05.

Matrigel cellular invasion assay showed that transient *CS* silencing inhibited SKOV3 and A2780 cell invasiveness (*P*<0.05, *P*<0.05) (Fig. 4A). Migration assay showed that *CS* silencing reduced migration ability of SKOV3 (*P*<0.05) and A2780 (*P*<0.01) (Fig. 4B). The expression of MMP2 was downregulated significantly (*P*<0.05) in *CS*-silenced cancer cells as shown by western blot. P-FAK was also downregulated, but there was no significant (*P*>0.05) (Fig. 4C). Studies of human epithelial carcinoma cell lines have demonstrated that Vimentin expression was induced in invasive cell lines [12], [13], we also found Vimentin protein level was decreased in *CS*-inhibited cancer cells (*P*<0.05) (Fig. 4C).

### CS silencing reduces drug resistance in ovarian cancer cells

**Figure 5:**
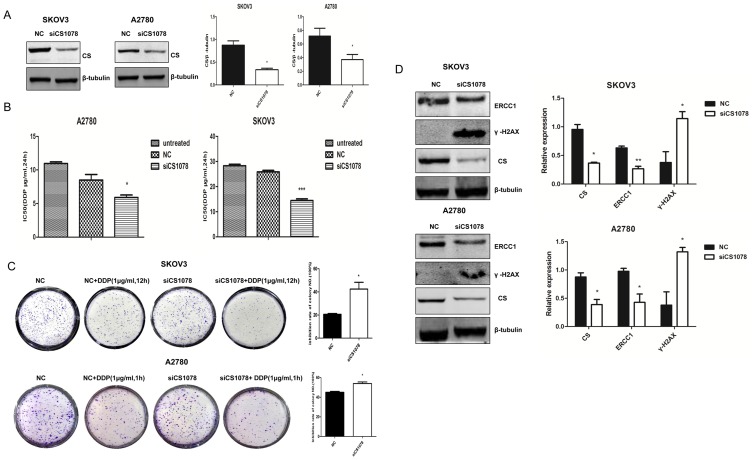
*CS* silencing increases drug sensitivity in ovarian cancer cells. (**A**) Protein level of CS was examined in *CS*-silenced SKOV3 and A2780 cells. (**B**) SKOV3 and A2780 cells were treated with *CS* siRNA or negative siRNA for 48 h, then cells were treated with different concentrations of DDP for another 24 h. Cell viability was measured after incubation with CCK8 for 1.5 h. (**C**) 48 h after *CS* siRNA transfection, SKOV3 and A2780 cells were treated with 1 µg/ml DDP for 12 h and 1 h, respectively. Then cells were plated in 6-well plates, colonies were stained and counted after incubation for 8 days. Results shown were representative of three independent experiments. (**D**) SKOV3 and A2780 cells were treated with *CS* siRNA for 48 h, ERCC1 and γ-H2AX protein levels were compared between NC and siCS1078 group with β-tubulin used as a loading control. Mean ± SEM. *P<0.05, **P<0.01 and ***P<0.001.

Given that CS was overexpressed in ovarian cancer, we further investigate the effect of CS on drug resistance of ovarian cancer cells. We transiently transfected siRNA targeting CS in SKOV3 and A2780 cells, CS expression was greatly reduced (*P*<0.05) (Fig. 5A). 48 h after transfection, cells were treated with DDP for 24 h and then CCK8 assay was used to examine cell viability. IC50 was downregulated in *CS*-inhibited SKOV3 and A2780 cells compared to the control after DDP treatment for 24 h (*P*<0.001, *P*<0.05) (Fig. 5B). Colony formation assay was performed at 12 h and 1 h post-cisplatin treatment respectively. Treatment with *CS* siRNA and DDP also resulted in significant reduction in SKOV3 and A2780 cell numbers as measured by colony formation assay (*P*<0.05) (Fig. 5C). Additionally, we had examined knockdown of CS could lead to increased apoptosis after DDP treatment by western blot. As shown in Fig. 3D, cleaved caspase 3 was enchanced after co-treated with *CS* siRNA and DDP. 48 h post-transfection, ERCC1, which contributes to platinum drug resistance by promoting DNA repair, was decreased (*P*<0.05), and γ-H2AX, which is related to cell apoptosis and drug resistance, was also increased (*P*<0.05) (Fig. 5D). These results showed *CS* silencing could improve sensitivity of ovarian cancer cells to DDP.

### Gene expression profile related to drug resistance, apoptosis, and autophagy changes upon CS knockdown

**Figure 6:**
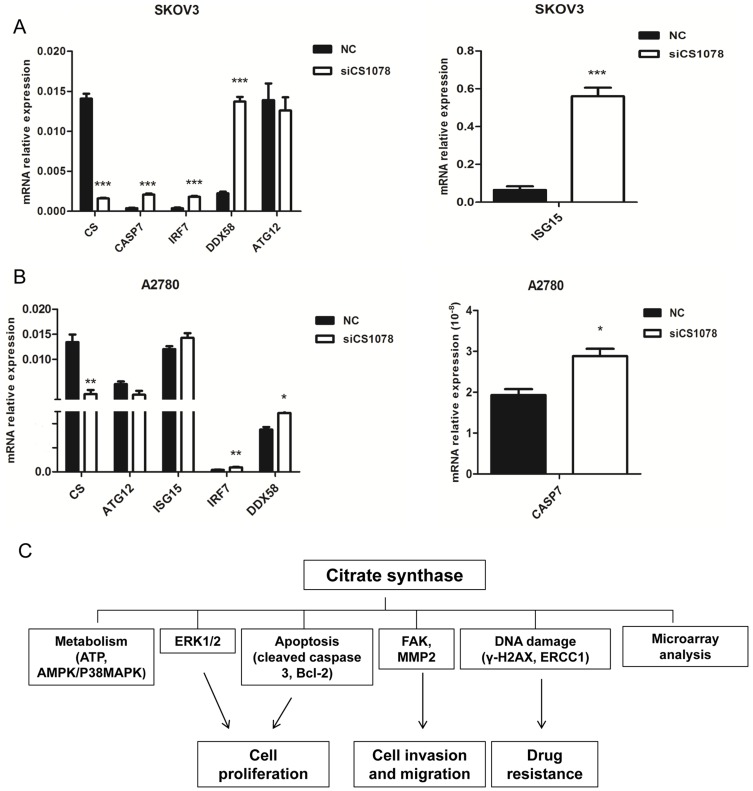
Gene expression profile related to drug resistance and apoptosis in SKOV3 and A2780 cells after *CS* silencing. (**A, B**) Ovarian cancer cells were treated with siRNA for 48 h, total RNA was extracted for gene expression analysis. Expression of *CASP7 (CASPASE7), IRF7 (*interferon regulatory factor 7), *DDX58 (*DEAD (Asp-Glu-Ala-Asp) box polypetide 58*)*, and *ISG15 (IFN*-stimulated gene 15*)* was increased significantly whereas *ATG12 (*autophagy related 12*)* expression was decreased in SKOV3 and A2780 cells after *CS* knockdown. *β-actin* was used as an internal control gene. Mean ± SEM. *P<0.05, **P<0.01 and ***P<0.001. (**C**) The diagram was shown to identify potential signaling pathways modulated by CS.

To gain insight into the effects of *CS* silencing, RNA derived from SKOV3 cells transfected with CS siRNA and NC siRNA was hybridized to Affymetrix HG-U133 Plus 2.0 microarray chips. After background subtraction and data normalization, 133 differently expressed genes were identified by preliminary screening using a cutoff value of 2-fold upregulation or downregulation. In order to validate these results, SKOV3 cells were treated with siRNA oligonucleotides for 24 h and mRNA expression of five genes related to apoptosis, metastasis and drug sensitivity – *CASP7, IRF7, DDX58, ISG15*, and *ATG12* – was analyzed by real-time PCR. Expression of the *CASP7, IRF7, DDX58*, and *ISG15* was increased significantly when *CS* was silenced in SKOV3 and A2780 cells whereas *ATG12* mRNA level was slightly decreased (*P*<0.05) (Fig. 6A, B).

## Discussion and Conclusion

Defect in the enzymes of the TCA cycle has recently been in the spotlight of the field of oncology [14]. CS catalyzes the first committed step of the TCA cycle and has been reported to be upregulated in several types of cancers [4], [5]. Consistent with these studies, we found that high level of CS was detectable in malignant ovarian tumors compared with benign ovarian tumors. Tumors are a heterogeneous mixture of cells containing tumor cells and varies types of non-carcinoma cells, such as leukocytes, endothelial cells, fibroblasts, myofibroblasts and bone marrow-derived progenitors [15], normal HOSE cultured from ovarian tissues was compared with SKOV3 and A2780 cancer cells. Expression of CS was upregulated in ovarian cancer cells compared with HOSE. These results suggested that CS might play an important role in human ovarian carcinoma.

A recent report showed that loss of CS in human cervical cancer cells induced morphological changes characteristic of an epithelial–mesenchymal transition (EMT), which has been shown to accelerate cancer cell metastasis and proliferation *in vitro* and *in vivo* [16]. In that study, *CS*-deficient cells exhibited severe defects in respiratory activity and marked decreases in ATP production, malignant progression was due to activation of EMT-related regulators [16]. In fact, many cancers exhibit the Warburg effect while retaining mitochondrial respiration [17]. ATP is an indicator of energy metabolism, ATP production was upregulated in CS-silenced SKOV3 and A2780 cells, but there was no significant difference, while AMPK/P38 MAPK was inhibited in *CS*-silenced cells. It might because there was compenastory mechanism to prompte TCA cycle in *CS*-silenced SKOV3 and A2780 cells. All eukaryotic cells possess multiple MAPK pathways, which coordinately regulate diverse cellular activities such as metabolism, gene expression, mitosis, motility, survival, apoptosis, and differentiation [18], and the ERK signaling is often upregulated in a diverse range of human cancers [19]. We discovered *CS* silencing could inhibit ovarian cancer cell growth accompanied with ERK phosphorylation downregulation. Cleaved caspase 3 is a key effector of apoptosis. Caspase 3 is present in healthy cells as a 32-kDa proenzyme that is cleaved to form the active heterodimer (17/12 kDa) upon apoptosis [20]. Bcl-2 protein functioned to oppose the apoptosis pathway of programmed cell death [21], [22]. Over-expression of Bcl-2 has been shown to promote cell survival by suppressing apoptosis. Anti-apoptotic Bcl-2 was significantly decreased and cleaved caspase 3 was increased after treated with or without DDP in *CS*-silenced SKOV3 and A2780 cells in the study. These results revealed that inhibition of CS expression result in dysregulation of cell metabolism and proliferation.

ERK and p38 phosphorylation have been linked to breast cancer metastasis and drug resistance [23]. In current study, we found cell migration and invasion ability were inhibited in *CS*-silenced cells. Consistent with these results, p-FAK, MMP2 and Vimentin protein levels were downregulated in *CS*-inhibited cells. Fidler et al. reported that FAK and MMPs were associated with metastases [24], secretion and activation of MMP2 might largely be responsible for the mechanism of decreased motility, invasiveness and metastasis of these cells [25]. Vimentin is a predominant intermediate filament (IF) protein in mesenchymal cells [26], associated with the invasive phenotype and its expression is required for carcinoma cell motility [27]. Taken together, these data showed ovarian cancer cell invasion and migration were inhibited in *CS*-silenced cells.

Cisplatin-based chemotherapies are used as the first-line treatment for ovarian cancers. Although there is often a high responsiveness at first, the majority of patients eventually progress with platinum-resistant disease [28]. By measuring cell viability and apoptosis of these two cells lines in response to DDP *in vitro* treatment by CCK8 and western blot, we discovered that *CS* knockdown could increase chemosensitivity of SKOV3 and A2780 to DDP. Results from colony formation assay further confirmed the findings from cell viability and apoptosis. In order to explore the molecular mechansim, ERCC1 and γ-H2AX were examined. ERCC1 expression and platinum resistance has been reported in patients with ovarian [29], [30], gastric [31], bladder [32], colorectal [33], non-SCLC [34], [35] cancers and melanoma [36]. As a part of post-translational modifications during apoptosis caused by severe DNA damage, high expression of γ-H2AX is considered as an accurate indicator of apoptosis [37] and it is also related to drug resistance [38]. The DNA double-strand break (DSB) is a serious lesion that can initiate genomic instability, ultimately leading to cancer [39], [40]. A key component in DNA repair is the histone protein H2AX, which becomes rapidly phosphorylated on a serine four residues from the carboxyl terminus (serine c-4) to form γ-H2AX at nascent DSB sites [41]. In addition to being a cause of cancer, DSB induction is paradoxically an effective treatment for cancer. Many therapeutic agents act by introducing sufficient DSBs into cancer cells to activate cell death pathways [42]. In our study, a decrease in ERCC1 protein level and an increase in γ-H2AX protein level were found in the treated cancer cells, suggested possible mechanism of increased apoptosis after *CS* silencing. Our results showed upregulated apoptosis level in *CS*-inhibited cancer cells might contribute to the reduced cell proliferation capacity and the increased drug sensitivity.

Through microarray analysis and real-time PCR, we discovered that knockdown of *CS* led to *IRF7, ISG15, DDX58*, and *CASP7* expression being increased in SKOV3 and A2780 cells, whereas *ATG12* expression was decreased. *CASP7* encodes Caspase 7 and is involved in the caspase activation cascade responsible for the execution of apoptosis [43], [44]. *IRF7* encodes one of the interferon regulatory factors and is required in breast cancer cells to prevent immune escape-mediated bone metastasis [45]. Elevated expression of *IFN*-stimulated gene 15 (*ISG15*), which encodes a protein that antagonizes the ubiquitin/proteasome pathway [46], [47], has been shown to confer camptothecin (CPT) sensitivity in breast cancer cells in part by interfering with topoisomerase I (TOP1) downregulation. DEAD (Asp-Glu-Ala-Asp) box polypetide 58 (*DDX58*; also known as Retinoic acid-inducible gene-I, *RIG-I*) encodes a cytoplasmic pathogen recognition receptor that recognizes pathogen-associated molecular pattern (PAMP) motifs that differentiate between viral and cellular RNAs. The RIG-I pathway is tightly regulated and aberrant signaling leads to apoptosis, altered cell differentiation, inflammation, autoimmune diseases, and cancer [48]. Notably, *IRF7, ISG15*, and *DDX58* contribute to chemosensitivity in breast cancer [45], [46], [48] and *CASP7* encodes a proapoptotic protein that may make cancer cells more sensitive to chemotherapy. Finally, *ATG12* is the human homolog of a yeast protein necessary to form autophagic vesicles [49]. Given that treatment with DDP has been shown to enhance reactive autophagy [50], the downregulation of *ATG12* observed upon *CS* silencing in SKOV3 and A2780 cell lines is consistent with our observations of decreased drug resistance. The diagram was shown to clearly identify potential signaling pathways modulated by CS (Fig. 6C).

Taken together, these findings demonstrated that abnormally upregulated expression of CS was found in human ovarian carcinoma, modulating its expression can influence cell proliferation, invasion, migration, and chemosensitivity of SKOV3 and A2780 cells. We thus propose that CS inhibition represents a novel therapeutic intervention to improve the prognosis of patients with ovarian cancer by suppressing metastasis and overcoming resistance of chemotherapy. As CS takes part in a complicated network in the organism metabolism, how CS is regulated in human ovarian carcinoma needs to be further explored.

## Supporting Information

S1 Fig **Primary HOSE cells cultured from normal ovarian epithelium tissues.** (**A**) Primary HOSE cells with stromal cells (10×). (**B**) Primary HOSE cells with stromal cells (20×). (**C**) Monolayer of HOSE cells after cell sorting (10×). (**D**) The normal HOSE cells were sorted by FACS Aria cell sorting system with EpCAM conjugated with PE as an epithelium marker.
(DOCX)
