# Supplementary material for: Citrate Synthase Expression Affects Tumor Phenotype and Drug Resistance in Human Ovarian Carcinoma
Source: PLoS One. 2014 Dec 29;9(12):e115708. doi: 10.1371/journal.pone.0115708 (PMC4278743; doi:10.1371/journal.pone.0115708)
Supplement: S1 Fig — Primary HOSE cells cultured from normal ovarian epithelium tissues. (A) Primary HOSE cells with stromal cells (10×). (B) Primary HOSE cells with stromal cells (20×). (C) Monolayer of HOSE cells after cell sorting (10×). (D) The normal HOSE cells were sorted by FACS Aria cell sorting system with EpCAM conjugated with PE as an epithelium marker. (DOCX) [file pone.0115708.s001.docx]

**Supporting Information**

**
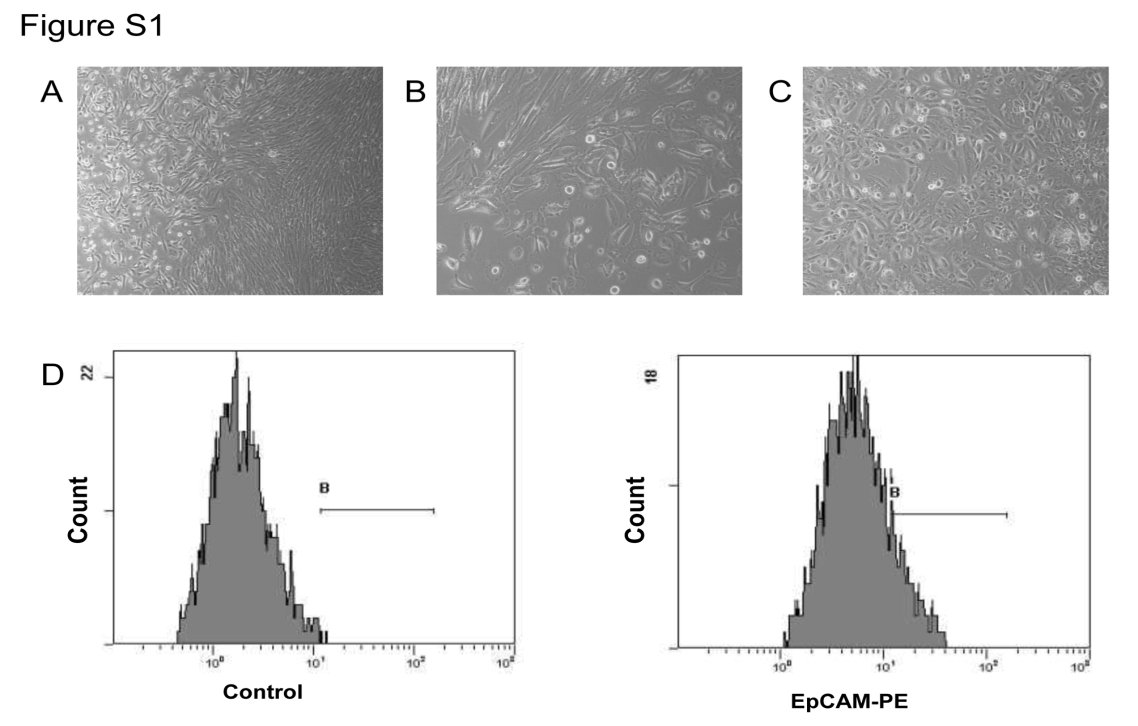
**

**Figure S1** Primary HOSE cells cultured from normal ovarian epithelium tissues. (**A**) Primary HOSE cells with stromal cells (10×). (**B**) Primary HOSE cells with stromal cells (20×). (**C**) Monolayer of HOSE cells after cell sorting (10×). (**D**) The normal HOSE cells were sorted by FACS Aria cell sorting system with EpCAM conjugated with PE as an epithelium marker.
